# Cancer-associated mutations in the canonical cleavage site do not influence CD99 shedding by the metalloprotease meprin β but alter cell migration *in vitro*

**DOI:** 10.18632/oncotarget.18966

**Published:** 2017-07-04

**Authors:** Tillmann Bedau, Neele Schumacher, Florian Peters, Johannes Prox, Philipp Arnold, Tomas Koudelka, Ole Helm, Frederike Schmidt, Björn Rabe, Marlene Jentzsch, Philip Rosenstiel, Susanne Sebens, Andreas Tholey, Stefan Rose-John, Christoph Becker-Pauly

**Affiliations:** ^1^ Unit for Degradomics of the Protease Web, Institute of Biochemistry, University of Kiel, 24118 Kiel, Germany; ^2^ Anatomical Institute, University of Kiel, 24118 Kiel, Germany; ^3^ Institute of Experimental Medicine, University of Kiel, 24118 Kiel, Germany; ^4^ Institute for Experimental Tumor Research, University of Kiel, 24118 Kiel, Germany; ^5^ Institute of Clinical Molecular Biology, University of Kiel, 24118 Kiel, Germany

**Keywords:** CD99, meprin β, proteolytic shedding, lung cancer, inflammation

## Abstract

Transendothelial cell migration (TEM) is crucial for inflammation and metastasis. The adhesion molecule CD99 was shown to be important for correct immune cell extravasation and is highly expressed on certain cancer cells. Recently, we demonstrated that ectodomain shedding of CD99 by the metalloprotease meprin β promotes TEM *in vitro*.

In this study, we employed an acute inflammation model (air pouch/carrageenan) and found significantly less infiltrated cells in meprin β knock-out animals validating the previously observed pro-inflammatory activity. To further analyze the impact of meprin β on CD99 shedding with regard to cell adhesion and proliferation we characterized two lung cancer associated CD99 variants (D92H, D92Y), carrying point mutations at the main cleavage site. Interestingly, ectodomain shedding of these variants by meprin β was still detectable. However the cleavage site shifted to adjacent positions. Nevertheless, expression of CD99 variants D92H and D92Y revealed partial misfolding and proteasomal degradation. A previously observed influence of CD99 on Src activation and increased proliferation could not be confirmed in this study, independent of wild-type CD99 or the variants D92H and D92Y. However, we identified meprin β as a potent inducer of Src phosphorylation. Importantly, we found significantly increased cell migration when expressing the cancer-associated CD99 variant D92H compared to the wild-type protein.

## INTRODUCTION

CD99 is a 32 kDa highly O-glycosylated type I transmembrane protein, which is expressed on cells of the hematopoietic system and on endothelial cells [1, 2]. The homophilic interaction of CD99 molecules on both immune and endothelial cell was identified as an essential step for transendothelial migration (TEM) of leukocytes from the blood vessel into surrounding tissue during inflammation [1, 3, 4]. Some studies showed that differential expression of CD99 is associated with several pathological conditions. For instance, high CD99 expression is a hallmark of Ewing sarcoma (EWS), a malignant mesenchymal bone tumor, and CD99 expression is routinely assessed as a prognostic marker [5, 6]. Conversely, loss of CD99 expression has been described in osteosarcoma, suggesting a tumor suppressor role of CD99 in this particular cellular context [7].

Recently, CD99 was found to be specifically cleaved by the metalloprotease meprin β [8, 9]. Importantly, ectodomain shedding of CD99 by meprin β and subsequent regulated intramembrane proteolysis by γ-secretase resulted in increased TEM of murine Lewis lung carcinoma (LLC) cells. The cleavage site in CD99 was identified within a highly conserved region, mainly consisting of negatively charged aspartate residues, which correlateswith the cleavage specificity of meprin β [10].

Meprin β is a zinc-dependent multidomain metalloprotease of the astacin family that acts as an ectodomain sheddase of type I transmembrane proteins at the plasma membrane but can also be shed itself by a disintegrin and metalloproteinase (ADAM) proteases [11]. The substrate repertoire of meprin β includes other type I transmembrane proteins such as the amyloid precursor protein (APP) [12–14] as well as soluble extracellular proteins such as mucin 2 (MUC-2) [15] or procollagen I [16]. Under physiological conditions, meprin β is expressed at the brush border membranes of the proximal kidney tubules and in the small intestine [11].

Interestingly, abnormal expression of CD99 and meprin β have been implicated in human tumors. For instance, meprin β mRNA levels are elevated in both primary and metastatic sites of pancreatic neuroendocrine tumors [17, 18]. Meprin β upregulation is thought to promote cancer cell migration and metastasis by cleaving extracellular matrix (ECM) proteins. In fact, meprin β promotes TEM of murine carcinoma cells thereby potentially increasing their metastatic potential [9].

We hypothesized that CD99 cleavage by meprin β plays a role in tumor development based on three reasons. Firstly, it is expressed on the endothelium and therefore might be involved in the extravasation of metastasizing tumor cells. Furthermore, it is upregulated in cancer suggesting that disruption of normal CD99 protein levels may be favorable for malignant transformation or tumor progression. And finally, meprin β deficient mice exhibit an accumulation of CD99 protein in the lung suggesting a role of meprins in CD99 homeostasis [9]. Here, we report on two CD99 point mutations identified in primary human lung tumors that directly affect the meprin β cleavage site. We speculated that the resulting amino acid exchanges may affect meprin β-mediated shedding and thereby alter functional properties such as cell proliferation and migration. Thus, we biochemically characterized the CD99 variants in cell based assays and furthermore, in a proof of principle approach, compared their influence on cell homeostasis and cell behavior to wild type (WT) CD99.

## RESULTS

**Figure 1:**
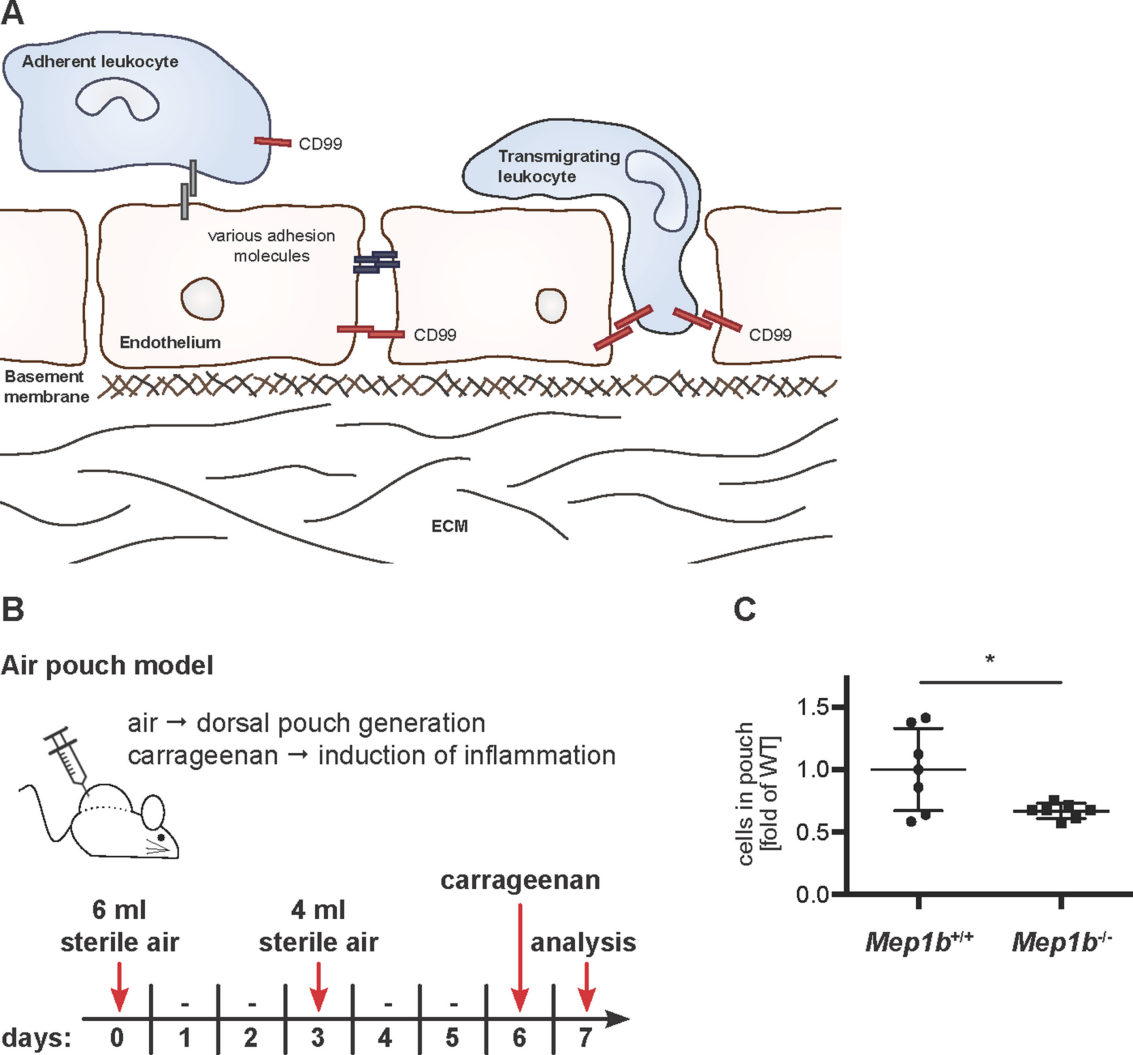
Meprin β is important for immune cell extravasation during acute inflammation (**A**) CD99 is crucial for TEM. Extravasation of leukocytes through endothelial layers requires specific interactions of adhesion molecules between both cell types. Chemokines and other biologically active factors secreted by mesenchymal cells induce leukocyte migration. Whether or not CD99 also plays a role in tumor cell extravasation during metastasis is unknown because these mechanisms are much more heterogeneous. (**B**) For an acute inflammation model, *Mep1b*^+/+^ and *Mep1b*^–/–^ mice were injected subcutaneously with sterile air. Six days later carrageenan was administered into the dorsal air pouch to induce an immune cell infiltration. After 24 h, the pouch was washed and the lavage fluid collected for further analysis. (**C**) Cells in the pouch lavage fluid were counted. *Mep1b*^–/–^ mice had significantly less infiltrated cells than *Mep1b*^+/+^ mice.

The CD99 adhesion molecule is an important regulator of immune cell extravasation (Figure 1A). We recently reported that CD99 ectodomain shedding by meprin β promotes TEM [9]. This was shown in an *in vitro* assay using bEnd.3 endothelial cells and Lewis lung carcinoma (LLC) cells as migrating cells. To demonstrate the impact of meprin β on cell migration *in vivo*, we now performed an acute inflammation model (air pouch), employing meprin β knock-out mice (*Mep1b*^–/–^) and compared the number of transmigrated inflammatory cells to wild-type control animals. After generating a dorsal pouch in mice, carrageenan was injected to stimulate immune response (Figure 1B). Indeed, in the absence of meprin β a significantly decreased number of infiltrated cells were observed in the pouch lavage compared to wild-type mice (Figure 1C, Supplementary Figure 1). This supports our previous *in vitro* findings and demonstrates that meprin β contributes to TEM *in vivo*, a crucial step in inflammation and potentially also in cancer metastasis. We now aimed to further elucidate the importance of meprin β mediated CD99 cleavage, as CD99 is one of the main cell adhesion molecules regulating cell extravasation [19].

**Figure 2:**
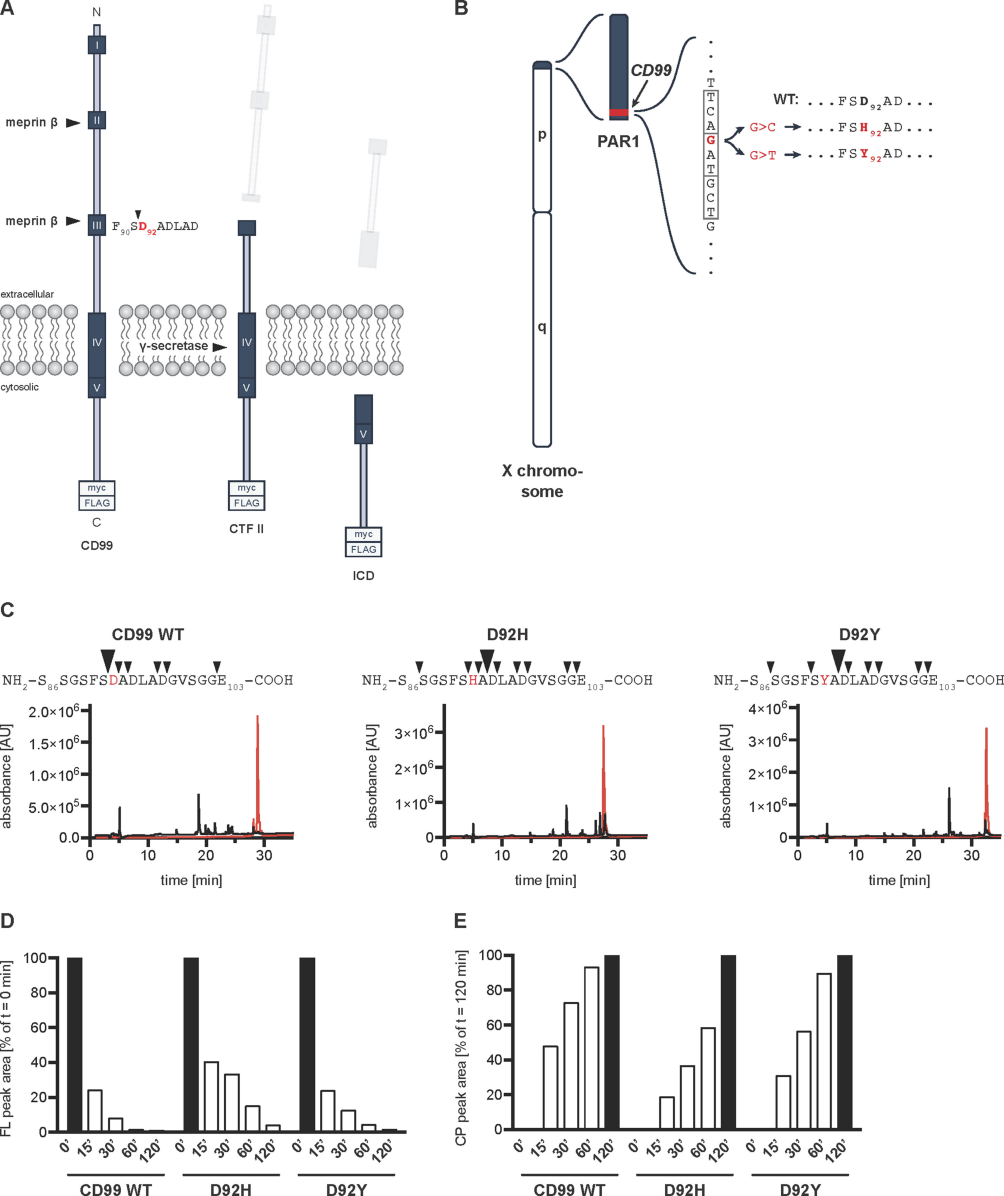
*CD99* lung cancer-associated mutations of Asp92 lead to a shift of the meprin β cleavage site (**A**) Schematic diagram of CD99 processing at the cell surface. Meprin β cleaves the full-length CD99 protein within highly conserved regions (HCR) II and III creating C-terminal fragments (CTF) I and II, respectively. CTF II is further processed by γ-secretase thereby releasing an intracellular domain (ICD) into the cytosol. For all further experiments, a C-terminally myc-/FLAG-tagged construct was used. HCRs are indicated by dark blue boxes with white Roman numerals. Arrowhead indicates meprin β cleavage site between Ser91 and Asp92 (red) affected by the mutations. C, C-terminus. N, N-terminus. (**B**) Genomic location of the lung cancer-associated *CD99* patient mutations. The human *CD99* gene (red) is located in the pseudoautosomal region (PAR) 1 at the end of the short arm of the X chromosome (blue). The mutations c.274G>C and c.274G>T (situated in exon 6) cause amino acid exchanges from Asp92 to His92 and Tyr92, respectively, thereby leading to loss of negative charge at the P1’ position, which is preferred by meprin β. p, p arm. q, q arm. (**C**) RP-HPLC chromatogram of synthetic peptides representing CD99 WT, D92H, and D92Y. Peptides spanning the meprin β cleavage site around Asp92 were analyzed alone (red graph) or after incubation with 15 nM recombinant meprin β for 1 h (black graph). Collected elution fractions were analyzed by MALDI-TOF MS and several cleavage sites were identified within the peptide sequences (black arrowheads). AU, arbitrary units. (**D**) Quantification of the decreasing FL peak areas from (Supplementary Figure 3). (**E**) Quantification of the increasing cleavage product peak areas from (Supplementary Figure 3).

Of note, both meprin β cleavage sites identified in CD99 are located within amino acid sequence regions that are highly conserved across vertebrate species [9, 20, 21]. This suggests coevolution of protease and substrate and underlines the apparent functional relevance of these sequence motifs. The highly conserved regions (HCRs) II and III consist of the amino acid sequences (F/L)DLX(D/E)A(V/L) and (F/I)XDXDLXD, respectively (X: any amino acid), which renders them as ideal meprin β cleavage sites considering the preference of the protease for negatively charged amino acid residues (D/E) around the scissile bond [10] (Figure 2A).

### Human lung cancer-associated point mutations in *CD99* gene cause amino acid exchanges directly within the meprin β cleavage site

In the BioMuta databank [22] two missense mutations in the *CD99* gene are annotated that directly modify the meprin β cleavage site. These variants are c.274G>C and c.274G>T, which cause amino acid (AA) substitutions of Asp92 towards His92 (p.D92H) or Tyr92 (p.D92Y), respectively (Figure 2B). Both mutations were detected in primary tumor samples of two male patients suffering from lung squamous cell carcinoma (p.D92H) or lung adenocarcinoma (p.D92Y). Asp92, found at the P1’ position, is the preferred cleavage site for meprin β resulting in a C-terminal fragment (CTF) II, which is further processed by γ-secretase [9] (Figure 2A). We hypothesized that loss of the negatively charged and preferred Asp92 would alter meprin β-mediated cleavage of CD99 and subsequent γ-secretase processing with possible impact on cancer cell biology.

### Peptides representing CD99 mutations are cleaved *in vitro*, however with a different cleavage site and kinetics

In order to assess the ability of meprin β to cleave the CD99 mutants *in vitro*, we initially analyzed synthetic peptides spanning the Asp92 cleavage site (Ser86-Glu103), containing either the WT or mutant (D92H and D92Y) AA sequence (Figure 2C). Indeed, all three peptides were cleaved by meprin β. Compared to the chromatogram of the full-length (FL) peptides alone, which eluted in one single peak, several peaks corresponding to cleaved peptides were detectable in the presence of recombinant meprin β after RP-HPLC (Figure 2C). The elution fractions were collected and subjected to MALDI-TOF MS (Supplementary Figure 2) to identify newly derived N-termini. Indeed, the preferred cleavage site at position Asp92 (WT, big arrowhead) was barely used anymore in case of the mutants with His or Tyr at this particular position. Instead, the main cleavage site shifted to Asp94, two AA further C-terminal (Figure 2C, big arrowheads). Also, both mutants were cleaved at other AA, thereby generating additional minor cleavage products (Figure 2C, small arrowheads). Furthermore, we determined cleavage kinetics by incubating the peptides with meprin β for 15, 30, 60, and 120 minutes (Supplementary Figure 3). By calculating the integral of the decreasing FL peak and the increasing main cleavage product peak, we quantified the cleavage process over time (Figure 2D and 2E, respectively). Interestingly, substitution of only Asp92 markedly changed the cleavage kinetics for the D92H mutant. The D92H peptide was processed much slower than the WT peptide. After 15 min, the FL peak area still amounted to 40% of the initial area while for the WT peptide the area was already reduced to 24% (Figure 2D). The difference was even more pronounced after 30 min: 33% compared to 8%. These data are in line with a slower increase of the cleavage product peak area (Figure 2E). For the D92Y peptide, however, the cleavage kinetics were similar to the WT peptide.

### CD99 mutants show different posttranslational modifications, but are equally processed by meprin β at the cell surface

**Figure 3:**
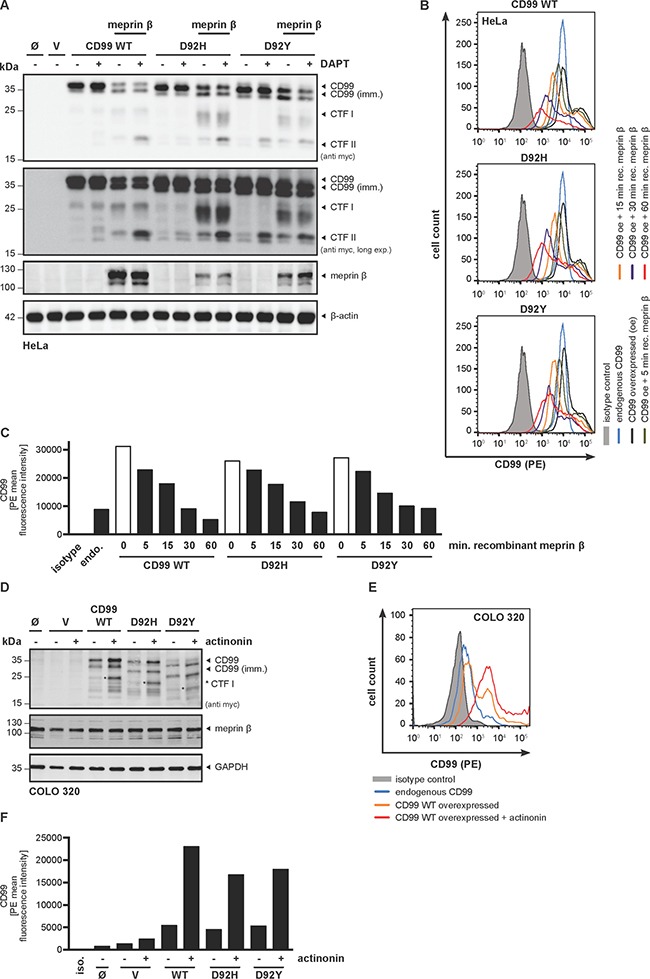
Meprin β-mediated shedding of CD99 variants induce regulated intramembrane proteolysis by γ-secretase (**A**) HeLa cells were transfected with CD99 WT, D92H or D92Y, with or without meprin β. Cell lysates were analyzed by Western blot using a specific meprin β or anti-myc antibody. β-actin served as loading control. For accumulation of γ-secretase dependent cleavage products the specific inhibitor DAPT (0.6 μM, 18 h prior to cell lysis) was applied. (**B**) To investigate cellular localization of CD99 and meprin β interaction we applied recombinant active meprin β (25 nM) to CD99-overexpressing HeLa cells and analyzed surface localization by flow cytometry. Here, a time-dependent decrease of the signal for CD99 WT and the cancer associated variants at the plasma membrane was observed. Signal specificity was verified by analysing CD99 overexpressing HeLa cells with IgG2a PE isotype control antibody. (**C**) Quantification of (B) using mean values of PE signal. Data represent PE mean values of one of three independent experiments. (**D**) COLO 320 cells, constitutively expressing high levels of endogenous meprin β, were transfected with CD99 WT, D92H or D92Y and treated with the meprin β inhibitor actinonin. GAPDH served as a loading control. Asterisks indicate CTF I fragments. (**E**) To analyze inhibition of endogenous meprin β with regard to CD99 shedding, COLO 320 cells were transfected with all CD99 variants and surface localization was measured by flow cytometry. (**F**) Quantification of (E) using mean values of PE signal. Data represent PE mean values of one of three independent experiments.

To study the CD99 mutants *in cellulo*, we generated C-terminally myc-/FLAG-tagged expression constructs, transfected HeLa cells, and analyzed cell lysates by Western blot (Figure 3A). CD99 WT appeared as two distinct bands: an intense upper one at around 35 kDa proposed to correspond to the fully glycosylated, properly folded, membrane-bound protein; and a weaker band at around 30 kDa corresponding to a non-or partially-glycosylated (immature) form. In contrast, expression of the D92H and D92Y variants revealed additional bands below 30 kDa and a shift of the full-length protein towards the 30 kDa form was observed. Co-expression of all CD99 variants with meprin β resulted in the generation of CTFs of similar size. Of note, a diffuse smear over a broad molecular weight range was observed in Western blots when the two CD99 mutants and meprin β were co-expressed. This indicates that more cleavage sites are used and more degradation is taking place, which correlates with the additional cleavage sites identified *in vitro* (Figure 2C). Addition of the γ-secretase inhibitor DAPT led to accumulation of CTF II in all cases (Figure 3A) as previously observed for WT CD99 [9]. To validate the cleavage sites identified *in vitro* in a cellular context, we excised immune-precipitated CD99 CTFs II from a Coomassie-stained SDS gel and performed LC-ESI MS/MS (Supplementary Figure 4). Indeed, we could confirm that, in case of the mutants, the cleavage site used by meprin β to generate CTF II shifts from Asp92 to Asp94 as observed in the peptide assays (Figure 2C). To analyze the ability of soluble shed meprin β to cleave the CD99 mutants, we applied recombinant meprin β (the soluble ectodomain containing the active protease domain) time-dependently to CD99-overexpressing HeLa cells and monitored the CD99 expression on the cell surface by flow cytometry (Figure 3B). Quantification shows that after 1 hour almost all CD99 was shed from the cells (Figure 3C). Remarkably, contrary to the *in vitro* kinetics (Figure 2D), there was no obvious difference in shedding kinetics between CD99 WT and the two mutants *in cellulo* (Figure 3C).

### CD99 mutants are constitutively shed by endogenous meprin β in COLO 320 cells

We used a human colorectal adenocarcinoma cell line (COLO 320) with high endogenous expression of meprin β to rule out the possibility that differences in CD99 cleavage were merely an artifact of meprin β overexpression. We therefore transfected COLO 320 cells with the different CD99 constructs and analyzed cell lysates by Western blot, which confirmed a similar cleavage pattern compared to HeLa cells (Figure 3D). Indeed, application of the meprin β inhibitor actinonin [23] resulted in accumulation of CD99 full-length protein indicating decreased ectodomain shedding of the adhesion molecule (Figure 3D). This was further confirmed by flow cytometry where an accumulation of CD99 protein was observed at the cell surface after treatment with actinonin (Figure 3E, 3F).

**Figure 4:**
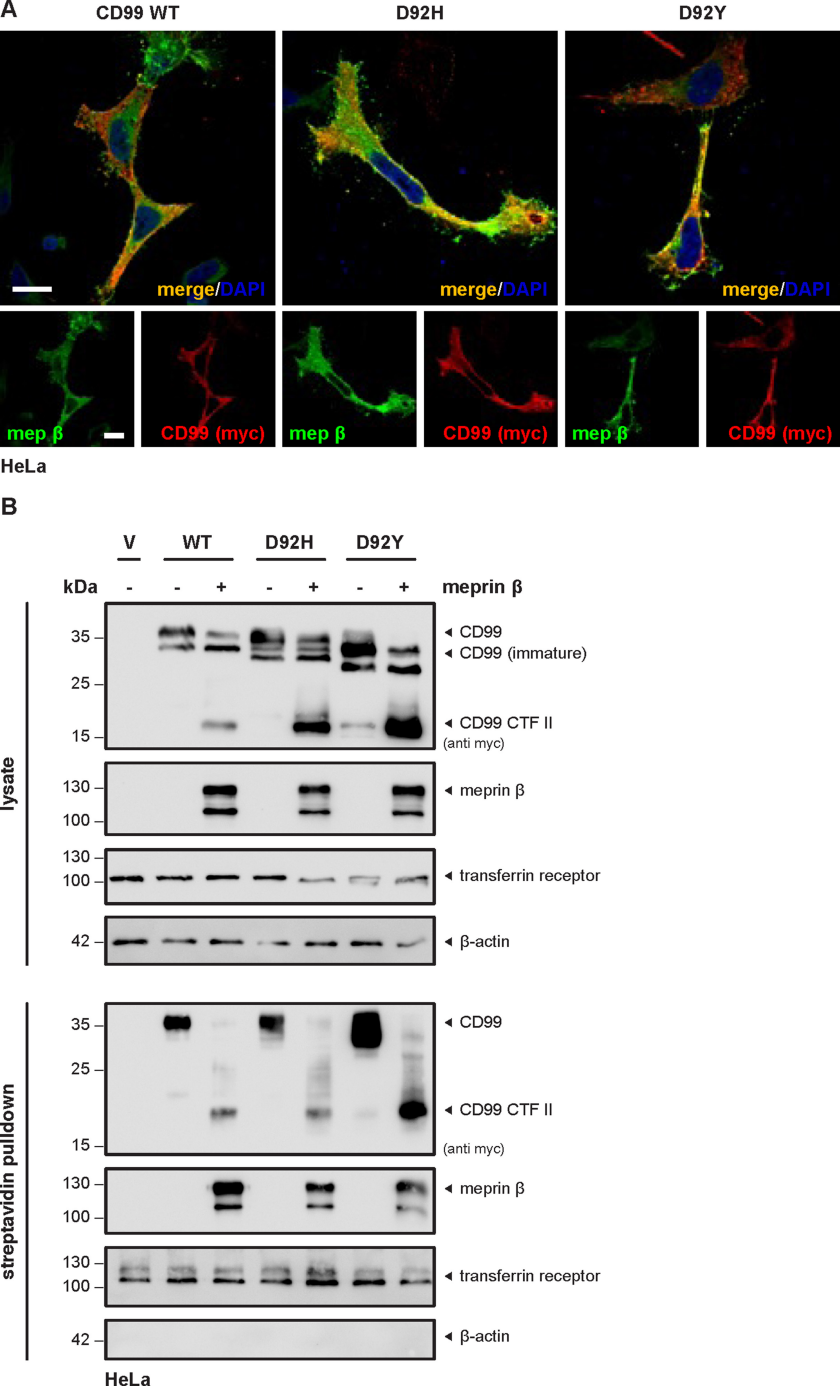
Only full-length mature CD99 variants are present at the cell surface (**A**) HeLa cells transfected with CD99 WT, D92H or D92Y and meprin β showed co-staining of protease and substrate at the cell surface using confocal fluorescence microscopy. Of note, more intracellular staining was observed for the cancer-associated variants compared to CD99 WT. Bar = 20 μm. (**B**) Cell surface proteins of HeLa cells transfected with CD99 WT, D92H or D92Y and meprin β were labelled by primary amine biotinylation, pulled down using streptavidin sepharose beads, and analyzed by Western blot. When coexpressed with meprin β, CTF II fragments appeared for all CD99 variants. For accumulation of γ-secretase dependent cleavage products the specific inhibitor DAPT (0.6 μM, 18 h prior to cell lysis) was applied. Transferrin receptor served as control for correct biotinylation.

We further investigated the subcellular localization of meprin β and the two CD99 variants by confocal laser scanning microscopy and could demonstrate colocalization at the cell surface (Figure 4A). Of note, expression of D92H and D92Y showed a stronger intracellular signal compared to CD99 WT. To analyze whether the actual proteolytic cleavage event also takes place at the cell surface, we additionally performed a biotinylation assay of surface proteins and analyzed these samples by Western blot. As already published for CD99 WT [9], not only the full-length CD99 protein but also CTF II could be detected in the biotinylated and pulled-down fraction in case of both mutants (Figure 4B), thus demonstrating that cleavage must take place at the cell surface. Interestingly, all proteins corresponding to the lower-migrating full-length bands between 22 and 30 kDa usually present in lysate fractions upon CD99 single transfection, were not precipitated by streptavidin. Therefore, the immaturity of these proteins (misfolding or incomplete glycosylation) most likely accounts for their lower molecular weight and intracellular accumulation. It is also noteworthy that the intensity of the full-length band at 35 kDa is completely restored or even higher than CD99 WT levels for both mutants when compared to the non-precipitated lysate control.

### Proteasomal inhibition leads to intracellular accumulation of D92H CTFs

**Figure 5:**
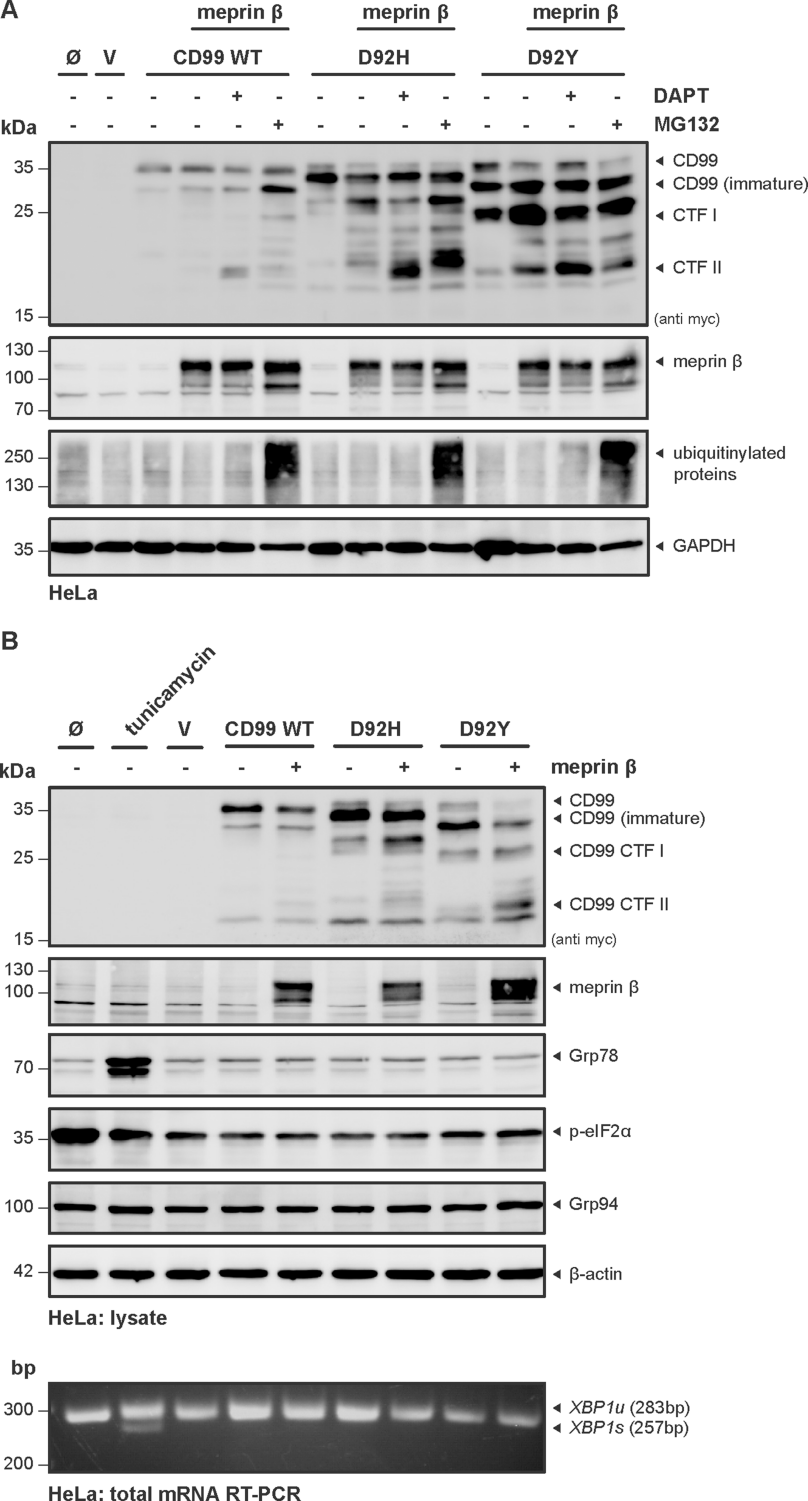
Misfolding and proteasomal degradation of Cd99 WT, D92H and D92Y does not induce ER-stress (**A**) HeLa cells were transfected with CD99 WT, D92H or D92Y, with or without meprin β. DAPT was used to inhibit γ-secretase activity and MG132 to prevent proteasomal degradation. Cell lysates were analyzed by Western blot using a specific meprin β or anti-myc antibody. Efficacy of MG132 treatment was validated by visualizing accumulation of ubiquitinylated proteins. GAPDH served as a loading control. (**B**) HeLa cells were transfected with CD99 WT, D92H or D92Y, with or without meprin β and cell lysates were analyzed by Western blot using anti-myc and specific meprin β antibodies. Additionally, ER stress-sensitive proteins were analyzed. mRNA of the same cells was isolated and analyzed for *XBP1* splicing by RT-PCR. In both cases tunicamycin, an inductor of ER stress, was used as positive control. *XBP1u*, unspliced *XBP1*; *XBP1s*, spliced *XBP1*.

In order to examine whether the different immature forms and the CTFs originating from cleavage of the CD99 variants cause cellular stress and initiate proteasomal activity we added the proteasome inhibitor MG132 to transfected HeLa cells and subsequently analyzed cell lysates by Western blot. There was only minor accumulation of CD99 CTFs when the WT construct was expressed together with meprin β (Figure 5A). However, upon co-transfection of D92H with meprin β, significant accumulation of two CTFs migrating slightly higher than CTF II was detected. This again indicates that this mutant is prone to more unspecific cleavage, thus generating CTFs, which are potentially detrimental for the cell and thus should be discarded.

### CD99 mutants do not induce ER stress

A quality control mechanism termed unfolded protein response (UPR), or ER stress, can sense misfolded proteins within the ER, which leads to a halt of translation, supports folding by upreglation of chaperones, but also enables degradation of excessive misfolded proteins. Ultimately, if the state is not resolved, the UPR may also lead to induction of programmed cell death. Based on the altered Western blot pattern of the CD99 variants, we hypothesized that these proteins might not be properly folded during synthesis and thus may trigger the UPR machinery. To address this, we analyzed cell lysates and total mRNA from HeLa cells for potential effects of the mutations in the presence or absence of meprin β. As readout for UPR induction, we used several molecular markers (Grp78, p-eIF2α, Grp94, XPB1 splicing) that have been shown to be sensitive for ER stress (Figure 5B). As a positive control, tunicamycin was used, which is known to induce ER stress via inhibition of proper glycosylation. Indeed, in the tunicamycin-treated sample induction of Grp78 and XBP1 splicing was evident. However, we could not detect significant alterations of these markers in the cells transfected with CD99 D92H and D92Y, indicating that the mutants do not induce ER stress.

### Meprin β induces Src phosphorylation independent of CD99

It was reported that CD99 attenuates Src kinase activity thereby increasing tumor malignancy and metastasis [24]. To investigate this in terms of proteolysis, we analyzed the impact of CD99 cleavage by meprin β with regard to Src phosphorylation.

**Figure 6:**
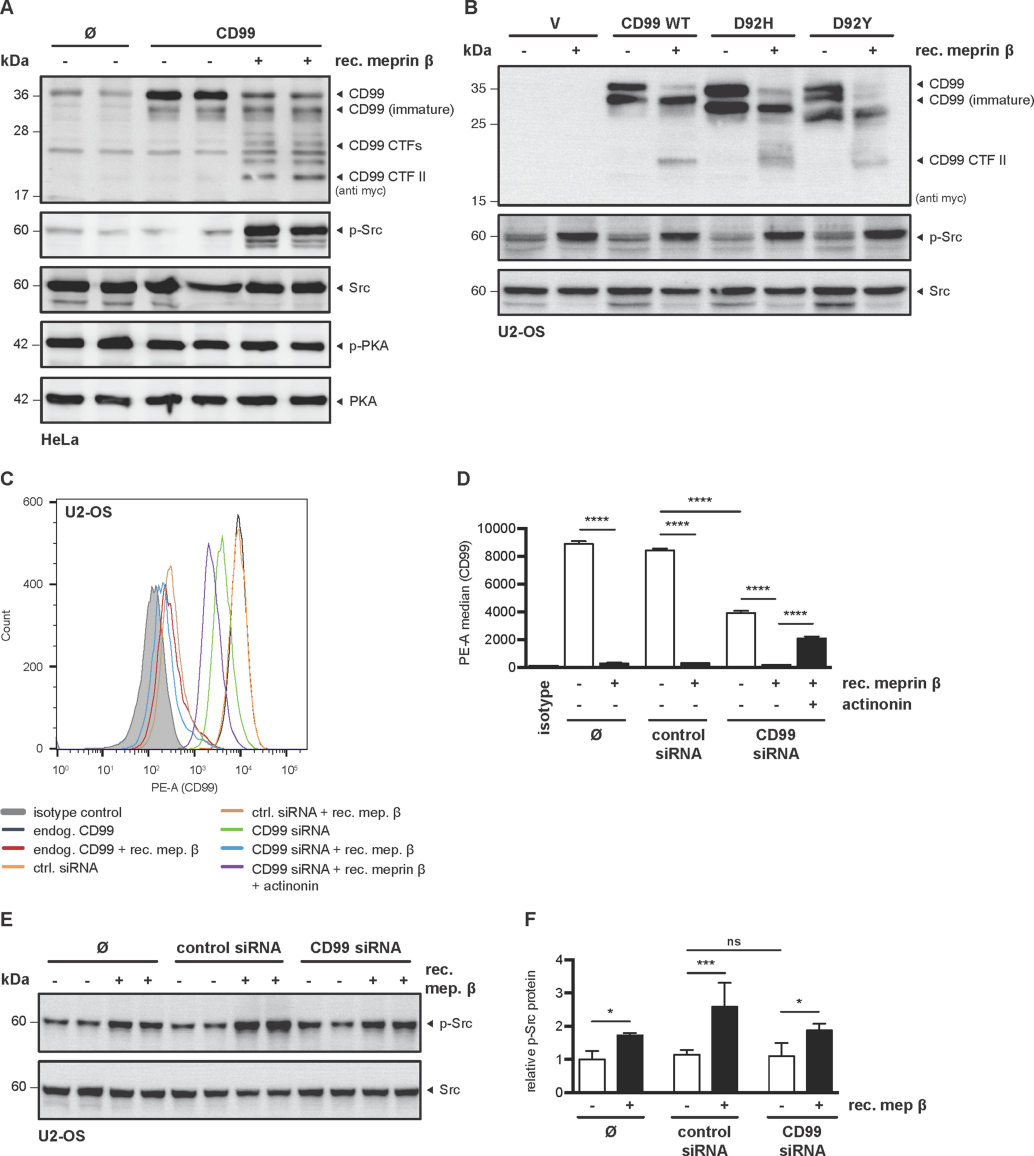
Meprin β induces Src phosphorylation independent of CD99 (**A**) HeLa cells were transfected with CD99, incubated with recombinant meprin β (25 nM) for 1 h, and lysates were analyzed for p-Src and p-PKA by Western blot. (**B**) To further test if Src activation is CD99-dependent, U2-OS were transfected with CD99 WT, D92H or D92Y in the presence or absence of meprin β (25 nM, 1 h) and analyzed by Western blot. (**C**) siRNA-mediated knockdown of endogenous CD99 in U2-OS cells was validated by flow cytometry. Knockdown specificity was validated by non-targeting siRNA. Incubation with recombinant meprin β (25 nM, 1 h) revealed shedding of CD99. Flow cytometry signal specificity was validated by using isotype control. (**D**) Quantification from (C) of cell surface CD99 in U2-OS cells by flow cytometry after siRNA transfection and application of recombinant meprin β. Almost complete loss of CD99 was observed after meprin β incubation, which could partially be rescued by adding the inhibitor actinonin (10 μM, 1 h). About 50% knockdown efficiency of CD99 was achieved by transfection with specific siRNA. Data represent mean of PE median values ± SD of technical duplicates from (C) and represent one of three individual experiments. ANOVA; ****p < 0.0001. (**E**) Cell lysates were produced from samples in (C) and then analyzed by Western blot. Only meprin β treated cells showed increased p-Src, whereas CD99 knockdown had no obvious effect. (**F**) Quantitative analysis of p-Src signal intensity in relation to Src from (E) calculated by Image J (version 1.49). Data represent mean p-Src/Src ratio ± SD of two individual experiments with technical duplicates as shown in (E). ANOVA; ***p < 0.001; *P < 0.05.

Therefore, HeLa cells were transfected with CD99 WT in the presence or absence of recombinant meprin β and p-Src was analyzed by Western blot (Figure 6A). Surprisingly, no obvious change in p-Src was detected when CD99 alone was expressed. However, incubation with active meprin β resulted in strong increase of p-Src, but not p-PKA, which is also thought to be involved in CD99 signaling [25]. In order to compare our experiments with the previously published data [24], we additionally transfected U2-OS (osteosarcoma) cells with CD99 WT or the cancer associated variants D92H and D92Y. However, application of meprin β resulted in enhanced p-Src, independent of CD99 (Figure 6B). Since we could not observe the published effect on p-Src, we considered that overexpression of CD99 might to some extent interfere with Src activation. Hence, we performed a knock-down of endogenous CD99 by siRNA. The specific siRNA-mediated decrease in CD99 cell surface expression was efficient (˜55%) as analyzed by flow cytometry (Figure 6C, 6D). Remarkably, incubation of U2-OS cells with 25 nM meprin β resulted in almost complete loss of CD99 at the plasma membrane and could partially be rescued through application of the meprin β inhibitor actinonin (Figure 6D). However, knock-down of CD99 in U2-OS cells did not result in changed p-Src levels (Figure 6E, 6F), confirming the data from CD99 overexpression. Again, application of recombinant meprin β strongly increased p-Src (Figure 6E, 6F).

### CD99 variant D92H causes increased cell migration

**Figure 7:**
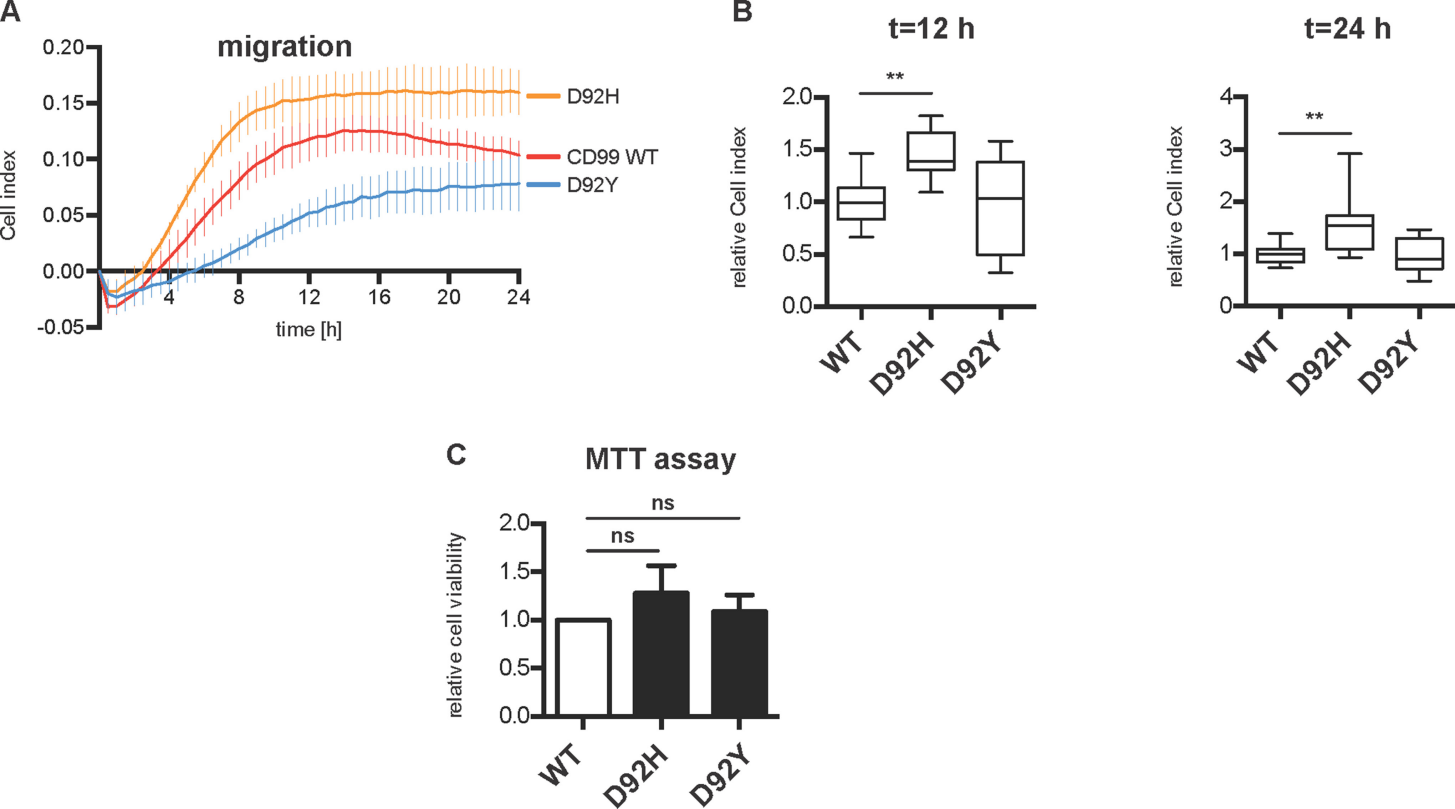
Overexpression of D92H variant increases cell migration in HeLa cells (**A**) HeLa cells were transfected with CD99 WT, D92H, or D92Y. After solubilization, 40,000 cells were added to xCELLigence microelectrode-equipped transwell chambers in triplicates and allowed to migrate for 24 h. Migration is monitored in real time as increase of impedance and is expressed as Cell index (CI), an arbitrary unit. (**B**) Quantification of CI values from (A) at time points 12 h and 24 h. Data represent mean ± SD of triplicates of four (CD99 WT and D92H) or three (D92Y) individual experiments (relative to CD99 WT). D92H-overexpressing cells show increased migration at both time points. ANOVA; **P < 0.01. (**C**) Cell proliferation was analyzed using an MTT assay.

After showing that both CD99 variants can be shed by meprin β and that they have no influence on Src activation, we investigated their effect on cell proliferation and migration where changes are typically associated with cancer and metastasis. Transiently transfected HeLa cells were seeded into transwell chambers and allowed to migrate through a porous membrane for 24 hours. Migration was monitored in real time using the xCELLigence system and increases in electrical impedance are reflected in an arbitrary unit, the cell index (CI) (Figure 7A). CI values at 12 h and 24 h were used for quantification and show significantly increased migration of cells overexpressing the D92H variant compared to cells overexpressing the CD99 WT protein. Migration of D92Y-overexpressing cells was not altered (Figure 7B). Proliferation was analyzed using an MTT assay. Here, no significant difference in proliferation rate was observed for the CD99 variants compared to cells transfected with CD99 WT (Figure 7C).

## DISCUSSION

Upregulation of the metalloprotease meprin β and its substrate CD99 is associated with several types of human cancers suggesting functions in tumor development. A common step in inflammation and metastasis is cell extravasation through endothelial cell layers [19, 26]. We recently demonstrated that CD99 is a substrate of meprin β *in vivo* and that this cleavage event promotes TEM *in vitro* [9]. In this study, we confirmed the pro-inflammatory activity of meprin β in an acute inflammation model, where we observed significantly decreased numbers of infiltrated cells in meprin β knock-out mice compared to WT animals. Hence, we hypothesized that the proteolytic interaction of meprin β and CD99 is important for TEM in health and disease, and therefore investigated two CD99 point mutations, which were identified in two primary human lung tumors [22]. Since the mutations D92H and D92Y cause an exchange of the preferred Asp92 to a disfavored amino acid within the meprin β cleavage site, we investigated whether shedding and cancer-associated cell behavior such as proliferation and migration would be altered and may contribute to tumor development and metastasis. Our results show that the CD99 variants D92H and D92Y are still shed by meprin β, albeit with the cleavage site shifted to the adjacent C-terminal aspartate residues within the HCR III ((F/I)XD_92_XDLXD) of CD99. Nevertheless, cleavage kinetics for D92H were slower when compared to the other CD99 variants. Interestingly, both cancer associated mutations showed a different band pattern in Western blot analysis. The additional, slightly lower-migrating bands, could not be detected at the cell surface, but were partially sensitive to proteasomal inhibition. Thus, the two CD99 variants seem to be more prone to misfolding. A formal induction of ER stress, however, was not observed. Cell biological effects of D92H, but not D92Y, were observed when this protein was overexpressed in HeLa cells. Here, cell migration significantly increased in an *in vitro* model when compared to CD99 WT. Thus D92H could destabilize binding of the cancer cell to the primary tumor or increase tumor cell extravasation and thereby favor metastasis. For other cell adhesion molecules, such as CECAM-1 or PECAM-1, similar involvement in cancer cell metastasis was described [27]. Of note, a previously described effect of CD99 on Src activation [24] or any changes for the two variants D92H and D92Y could not be confirmed in this study. Instead, meprin β significantly increased Src phosphorylation, independent of CD99, which might be an important pro-tumorigenic activity of this enzyme and warrants further analysis.

In summary, our results show that i) the ability of meprins to cleave the cancer-associated CD99 variants is not largely impaired although the cleavage site shifted, ii) both, D92H and D92Y seem to be partially misfolded and are more prone to proteasomal degradation than CD99 WT, and iii) D92H causes significantly higher cell migration compared to CD99 WT.

## MATERIALS AND METHODS

### Cell culture and transient transfection

HeLa, COLO 320, and U2-OS cells were grown in Dulbecco's Modified Eagle Medium (DMEM) supplemented with 10% fetal calf serum (FCS) at 37°C and 5% CO_2_. For experiments involving treatment with recombinant meprin β, cells were cultured in serum-free medium during treatment to avoid protease inhibition by serum ingredients. Cells were transiently transfected at 60% confluency using polyethylenimine (PEI) and the following expression constructs: empty vector control (pcDNA3.1), human meprin β (both pcDNA3.1, kindly provided by Erwin Sterchi), human CD99 WT (pCMV6 myc-/FLAG-tagged, Origene, #RC204056), and D92H and D92Y (self-mutated from hCD99 WT construct). For siRNA knockdown experiments U2-OS cells were transfected with Silencer Select CD99 siRNA or Silencer Select Negative Control siRNA (#4392420 and #4390843, respectively, Thermo Fisher Scientific) using the transfection reagent INTERFERRin (Polyplus transfection).

### Site-directed mutagenesis

Human CD99 D92H and D92Y mutants were produced by site-directed mutagenesis with hCD99 WT myc-/FLAG-tagged construct serving as template DNA. The following primers were used: c.274G>C forward: 5′-cta gttcctccggtagcttttcacatgctgaccttgcggatggc-3′, c.274G>C reverse: 5′-gccatccgcaaggtcagcatgtgaaaagctaccggaggaac tag-3′; c274G>T forward: 5′-ctagttcctccggtagcttttcatat gctgaccttgcggatggc-3′; c274G>T reverse: 5′-gccatccgcaag gtcagcatatgaaaagctaccggaggaactag-3′. Primers were phosphorylated, then added to template DNA and DNA was amplified by Phusion Taq DNA Polymerase (ThermoFisher). Methylated template DNA was then digested by DpnI enzyme and the amplified DNA was ligated. Introduction of both point mutations was confirmed by DNA sequencing (GATC Biotech).

### Cell lysates, SDS-PAGE, and western blot

Cells were washed once with PBS, harvested using a sterile cell scraper, and centrifuged at 500 g for 10 min at 4°C. The cell pellet was resuspended in lysis buffer (1 mM EGTA, 5 mM TRIS, 250 mM saccharose, 1% Triton X-100, complete protease inhibitor (Roche), pH 7.4) and placed on ice for 30 min. For phosphoproteins a different lysis buffer was used: 137 mM NaCl, 20 mM TRIS, 1 mM Na_3_VO_4_, 1% NP-40, cOmplete proteinase inhibitor cocktail, PhosSTOP phosphatase inhibitor (both Roche), pH 8.0. Cell debris was removed by centrifugation at 13, 200 rpm for 15 min at 4°C and supernatant was removed. The protein concentration was determined using a BCA protein assay kit (ThermoFisher) and equal amounts of protein were mixed with 5X reducing sample buffer and denatured at 95°C for 10 min. Protein extracts were separated on 10% or 12% SDS-PAGE (130 V, 90 min) and afterwards transferred onto nitrocellulose membranes using tank blot procedure (800 mA, 2 h). Membranes were blocked in 5% milk in TBS-T or 5% BSA in TBS-T for phosphoprotein analysis for 1 h at room temperature and subsequently incubated with primary antibody in blocking solution at 4°C over night. The following primary antibodies were used: monoclonal anti myc (9B11, Cell Signaling, #2276), polyclonal anti meprin β (both selfmade, Pineda Antibody-Service), monoclonal anti polyubiquitinylated proteins (FK1, Enzo, #BML-PW8805), monoclonal anti Grp78 (C50B12, Cell Signaling, #3177), monoclonal anti p-EIF2α Ser51 (D9G8, Cell Signaling, #3398), polyclonal anti Grp94 (Cell Signaling, #2104), polyclonal anti Src (Cell Signaling, #2108), polyclonal anti p-Src Tyr416 (Cell Signaling, #2101), polyclonal anti PKA (Cell Signaling, #4782), polyclonal anti p-PKA Thr197 (Cell Signaling, #4781), polyclonal anti transferrin receptor (abcam, #ab84036) polyclonal anti actin (Sigma, #A2066), monoclonal anti GAPDH (14C10, Cell Signaling, #2118). After washing with TBS-T, membranes were incubated with horseradish peroxidase-conjugated secondary antibody for 1 h at room temperature. Membranes were then washed again and proteins were detected using the SuperSignal West Femto Maximum Sensitivity Substrate (ThermoFisher) on a LAS-3000 mini (Fujifilm) imaging system.

### Cell surface protein biotinylation

Transfected cells were cooled down to 4°C and washed with PBS-CM (0.1 mM CaCl_2_ and 1 mM MgCl_2_ in PBS). Biotin solution (1 mg/ml EZ-Link Sulfo-NHS-SS-Biotin (ThermoFisher) in PBS-CM) was added for 30 min at 4°C. Afterwards, cells were incubated with quenching buffer (50 mM TRIS, pH 8 in PBS-CM) for 10 min at 4°C and washed three times with PBS-CM. Cells were then harvested in PBS-CM and lysed (50 mM TRIS, 150 mM NaCl, 1% Triton X-100, 0.1% SDS, cOmplete protease inhibitor (Roche), pH 7.4). The total protein amount was determined using a BCA protein assay kit (ThermoFisher) and sufficient amount of lysate was taken for control Western blot analysis. The remaining lysate was incubated with washed High Capacity Streptavidin Agarose (ThermoFisher) for 1 h at 4°C to pull down biotinylated cell surface proteins. The agarose beads were washed three times with lysis buffer. Then, 80 μl 1X reducing sample buffer was added and samples were heated for 30 min at 60°C to separate precipitated proteins from agarose beads. Whole lysates and precipitated proteins were then analyzed by Western blot.

### Recombinant proteins

Recombinant meprin β was expressed in insect cells using the Bac-to-Bac Baculovirus Expression System (ThermoFisher) and purified as described previously [28]. For cleavage assays, cells were washed twice with PBS and recombinant meprin β was added in DMEM without FCS to circumvent protease inhibition by serum ingredients. For inhibition studies meprin β was preincubated with the inhibitor actinonin in DMEM for 15 min at room temperature prior to application to the cells.

### Peptide cleavage assays and HPLC analysis

Peptide cleavage assays were performed as described before [9]. In short, synthetic peptides were obtained from Genosphere Biotechnologies and analyzed by reversed-phase high-performance liquid chromatography (RP-HPLC). The peptide solution (500 μM) was either analyzed alone or after preceding incubation with recombinant meprin β (15 nM) at room temperature. The digestion was stopped after each timepoint by addition of 0.1% trifluoroacetic acid. Collection of eluting fractions corresponding to chromatogram peaks was performed for subsequent mass spectrometry-assisted peptide analysis.

### Identification of cleavage sites from HPLC fractions: MALDI mass spectrometry

A volume of 250 μl from each LC-fraction was dried via vacuum centrifugation to concentrate the sample. The dried samples were reconstituted in 25 μl of 3% ACN, in 0.1% TFA and 1 μl of the sample was then spotted onto a MALDI target in duplicate and left to air-dry. MALDI matrix (3 mg/ml of alpha-cyano-4-hydroxycinnamic acid) in 70% ACN, 0.1% TFA was then applied to the dried spots. MALDI MS/MS analysis was performed using an AB Sciex 5800 MALDI TOF/TOF mass spectrometer (ABSciex, Darmstadt, Germany). Measurement conditions were: reflectron positive ion mode; 2,000 laser shots per spot were acquired for MS spectra and a one point recalibration was performed using matrix peak at m/z 877.034. For MS/MS experiments, 2,500 shots were averaged with a pulse rate of 1,000 Hz. Precursor ions were separated by timed ion selection with 300 resolution window (FHWM). Peptides were fragmented either with post-source decay (PSD) or collision induced dissociation (CID) with deceleration to an energy of 1 kV. For CID experiments, ambient air was used as collision gas with medium pressure of 10–6 torr. Fragmentation spectra were analyzed with the “Ion fragmentation calculator” tool embedded in the Data explorer software 4.10 (AB Sciex) and manually inspected. Theoretical peptide masses and peptide mass deviations were calculated using Mmass (version 2.4).

### Identification of cleavage sites from cell lysates by SDS-PAGE, reductive dimethylationin-gel digestion and LC-MS/MS

CD99 C-term was immunoprecipitated from HeLa cell lysates transfected with myc/FLAG-tagged CD99 and untagged meprin β using an anti-FLAG antibody. The precipitate was separated by SDS-PAGE (12% gel) and stained by Coomassie. Bands, (Supplementary Figure 4A) according to signals detected in a control Western blot (Supplementary Figure 4B), were excised using a scalpel. Reductive dimethylation and in-gel digestion was performed as reported previously with minor modifications [16], i.e., digestion was carried out with 50 ng of chymotrypsin and 25 ng trypsin overnight in 10 mM HEPES buffer (2 mM CaCl_2_, pH 7.5) at 37°C.

LC-MS analyses were performed on an UltiMate 3000 RSL Nano/Cap System (Thermo, Bremen, Germany) coupled online to an OrbitrapQ Exactive mass spectrometer (Thermo). LC running conditions have been reported previously [29]. MS scans were acquired in the mass range of 300 to 2,000 m/z at a resolution of 70,000. The ten most intense signals were subjected to HCD (higher collisional energy dissociation) using a dynamic exclusion of 15 s. MS/MS parameters: minimum signal intensity: 1000, isolation width: 3.0 Da, charge state: ≥ 2, HCD resolution: 15,000, Normalized collision energy of 25. Lock mass (445.120025) was used for data acquired in MS mode. HCD spectra were searched using Proteome Discoverer 1.4 (1.4.0.288, Thermo Fisher Scientific) with the Sequest-HT search algorithm against the reviewed and canonical human database with contaminants (ftp://ftp.thegpm.org/fasta/cRAP/) and different mutants of CD99 appended to the database (20,319 sequences). The following database search settings were used: MS tolerance; ± 10 ppm, MS2 Tolerance; 0.02 Da, enzyme specificity; none, fixed modification at lysine (dimethylation) and cysteine (carbamidomethylation) residues. Modification at the peptide N-terminus (dimethylation) was set as a variable modification.

### Immunocytochemistry

HeLa cells were analyzed by immunofluorescence as described before [9]. In short, cells were seeded onto coverslips, transfected, and fixed with paraformaldehyde. After blocking and permeabilization, primary antibodies (anti myc, 1:1000; anti meprin β, 1:500) were added over night at 4°C. Secondary antibodies (Alexa Fluor 488 and 594) were applied for 1 h at room temperature before mounting the coverslips on glass slides together with DAPI for nuclear staining. Images were then acquired with a confocal laser scanning microscope (FV1000, Olympus).

### Flow cytometry

CD99 surface expression was monitored by flow cytometry. Cells were detached using Accutase (Sigma-Aldrich), pelleted, resuspended in FACS buffer (1% BSA in PBS) and 10^6^ cells were incubated with phycoerythrin (PE)-labeled anti human CD99 antibody (BioLegend, #318008) or PE-labeled IgG2a isotype control antibody (BioLegend, #400214) for 30 min on ice. Cells were then pelleted, washed twice, and resuspended in FACS buffer. Flow cytometric analysis was carried out on a Becton Dickinson FACS Canto system. Data analysis was performed using FlowJo software, V10.1.

### RNA isolation and RT-PCR

Total RNA from HeLa cells was extracted using the NucleoSpin^®^ RNA isolation kit (MACHEREY-NAGEL) according to the manufacturer's instructions. Amount and quality of extracted RNA was determined with Nanodrop (Thermo Fisher Scientific) by the absorbance at 260 nm and by the ratio of 280/260 nm. Total mRNA was reverse transcribed using RevertAid RT Reverse Transcription Kit (Thermo Fisher Scientific) according to the manufacturer's instructions. The PCR reaction was prepared with the DreamTag DNA Polymerase kit (Thermo Fisher Scientific) according to the manufacturer's instructions and the reaction products were separated and visualised on a 2.5% agarose gel. The following primers were used: *hXBP1* 5′-TTACGAGAGAAAACTCATGGCC-3′ and 5′-GGGTCCAAGTTGTCCAGAATGC-3′.

### Migration assay

HeLa cells were transfected with different CD99 constructs, detached using a cell scraper, counted, and resuspended in DMEM, 1% FCS. The real-time migration assay was performed using the xCELLigence RTCA DP analyzer (ACEA Biosciences). A CIM-Plate 16 was assembled and equilibrated with DMEM, 1% FCS for 1 h. Then 40,000 cells were added and allowed to migrate through the microporous membrane for 24 h. Results are expressed as cell index (CI), a relative unit reflecting the electrical impedance measurement at the bottom of the microporous membrane.

### Cell viability assay

HeLa cells were transfected with human CD99 WT, D92H, D92Y or empty control vector. Cells were seeded in a 96 well plate at a density of 50,000 cells per well. Next day, 96 well plate was centrifuged to pellet the cells, medium was removed and exchanged with 100 μl fresh culture medium. To each well 10 μl of a 12 mM MTT (Sigma) solution were added and incubated for 2.5 h at 37°C. Upon addition of 100 μl SDS-HCl solution (10% SDS in 0.01 M HCl) to each well and mixture using a pipette, microplate was again incubated for 2.5 h at 37°C. Afterwards, each sample was mixed again and the absorbance was detected at 570 nm. Relative cell viability was calculated by setting empty vector transfected cells to 100%.

### Mice

The generation of *Mep1b*^–/–^ mice has been described previously [30]. Mice were kept under specific pathogen free conditions in isolated ventilated cages, on a 12 h light-dark cycle with food and water ad libitum. Our investigations were carried out in accordance to the Guide for the Care and Use of Laboratory Animals of the German Animal Welfare Act on protection of animals. All animal protocols were approved by the Central Animal Facility of the University of Kiel and the relevant German authorities.

### Air-pouch mouse model

The air pouch model was performed as described previously [31, 32]. Briefly, subcutaneous injection of 6 ml sterile air into the back of 9–11 week old mice lead to dorsal pouches, which were re-injected with 4 ml of sterile air 3 days later. On day 6, 1 ml of 1% carrageenan (Sigma-Aldrich) in sterile PBS was injected into the pouches to induce an inflammatory response. 24 h after induction of inflammation blood was collected submandibular, mice were killed by cervical dislocation and the pouches were washed with 3 ml of 2 mM EDTA/PBS. Lavage fluid was immediately stored on ice and cells counted using *Cellometer* automated cell counter. Aliquots of 500 000 pouch cells or blood diluted in 40 mM EDTA/PBS were used for FACS analysis. After blocking Fc-receptors with mouse Fc block CD16/32 mAb (Biolegend) cells were stained with fluorescence-coupled mAbs against CD45 (30-F11), CD115 (AFS98), CD11b (M1/70), Ly6G (1A8), Ly6C (HK1.4) (Biolegend) and CD99 (#FAB3905P) or respective isotype control (#IC108P) both purchased from R&D Systems. Cells were fixed with RBC Lysis/Fixation Solution (Biolegend) and analysed by flow cytometry (FACS Canto II, BD). Data were analysed using FlowJo software (V 10.1).

### Statistical analysis

All statistical analysis was performed using Graph Pad Prism 6 software for Student's t test or one way ANOVA followed by a Tukey multiple comparison post hoc test. Values are expressed as mean ± SD. The null hypothesis was rejected at P < 0.05 (*P < 0.05, **P < 0.01, ***P < 0.001).

## SUPPLEMENTARY MATERIALS FIGURES



## Abbreviations

TEM: transendothelial migration
ECM: extracellular matrix
UPR: unfolded protein response
HCR: highly conserved region
CTF: C-terminal fragment
