# Supplementary material for: Cancer-associated mutations in the canonical cleavage site do not influence CD99 shedding by the metalloprotease meprin β but alter cell migration in vitro
Source: Oncotarget. 2017 Jul 4;8(33):54873–88. doi: 10.18632/oncotarget.18966 (PMC5589627; doi:10.18632/oncotarget.18966)
Supplement: Supplementary file 1 [file oncotarget-08-54873-s001.pdf]

# Cancer-associated mutations in the canonical cleavage site do not influence CD99 shedding by the metalloprotease meprin β but alter cell migration *in vitro*

## SUPPLEMENTARY MATERIALS

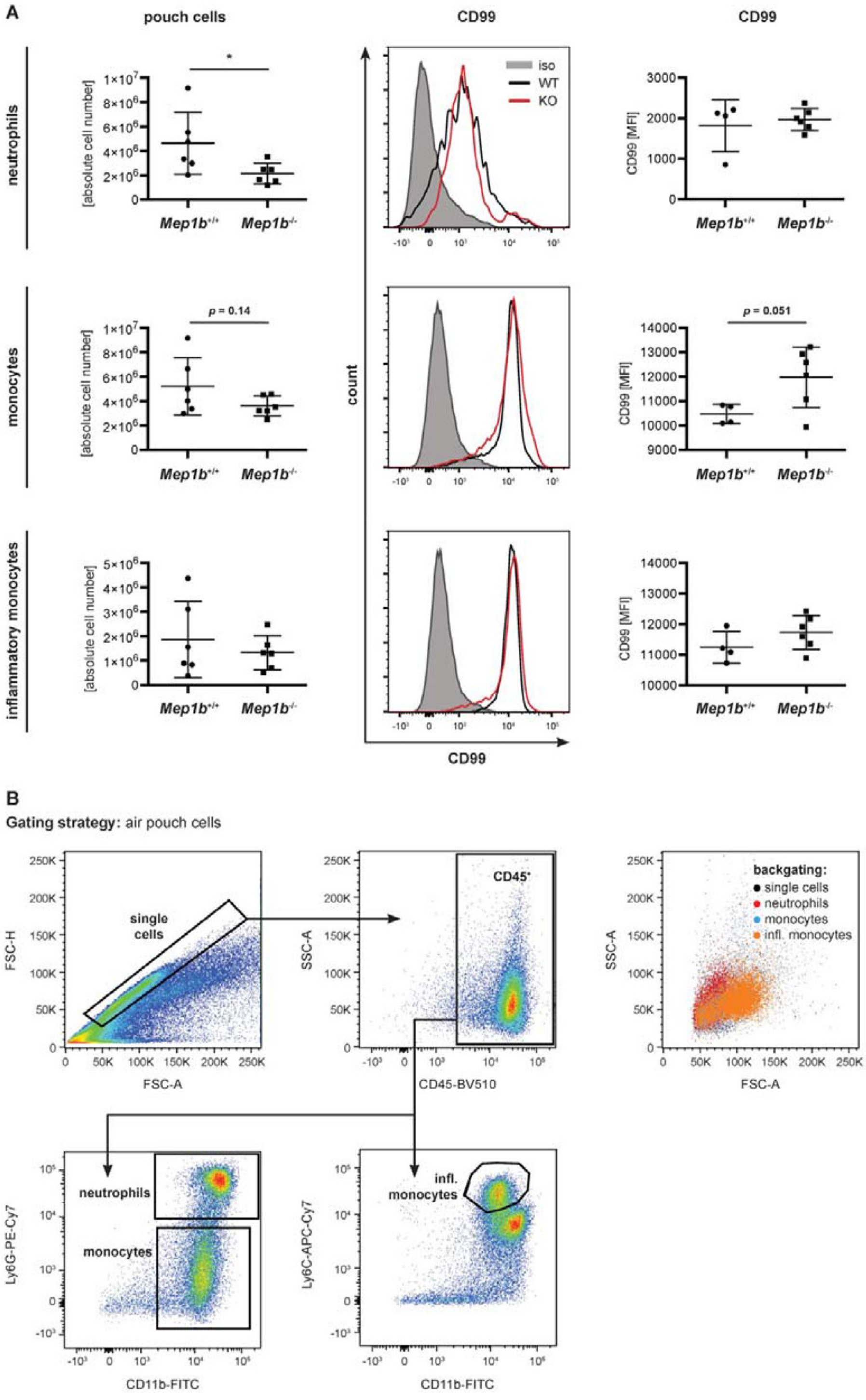


**Supplementary Figure 1: Flow cytometry analysis of blood from air-pouched mice.** (**A**) Pouch lavage fluid was analyzed by FACS. *Mep1b*$^{-/-}$ mice showed significantly lower neutrophils infiltrated into the air pouch cavity whereas monocyte numbers were not altered in comparison to *Mep1b*$^{+/+}$ mice (left panel). CD99 surface expression on infiltrated immune cells was not significantly different between *Mep1b*$^{-/-}$ and *Mep1b*$^{+/+}$ mice (middle panel). CD99 surface expression FACS histograms of *Mep1b*$^{-/-}$ and *Mep1b*$^{+/+}$ mice are

shown exemplarily (red and black line, respectively). PE mean fluorescence intensity was used for quantification (right panel). All values are expressed as mean ± SD. WT, wild-type; KO, knock-out. (**B**) Representative flow cytometry diagrams used for gating of air pouch mice immune cells. Pouch leukocytes were gated during FACS analysis as follows: single cells were gated from FSC-A/FSC-H plot. These cells were analyzed for CD45 expression and all CD45+ cells were subgrouped into neutrophils (CD11b+Ly6G+), monocytes (CD11b+Ly6Glow), and inflammatory monocytes (CD11b+Ly6C+). Proper gating was confirmed by backgating in a FSC/SSC plot (top row, right panel).

**A** Chromatograph of Peptide 2 (SSGSFSDADLADGVSGGE) and the peptides identified in the collected fractions

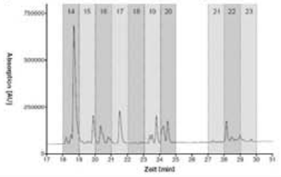


| Peptide – Fraction | AA | Sequence | Observed Mass (Na-H)$^+$ | Theoretical Mass (Na-H)$^+$ | Deviation (Da) |
|---|---|---|---|---|---|
| 2-14 | [1-6] | SSGSFS.D | 593.207 | 593.2183 | 0.0113 |
| 2-15 | [1-7] | SSGSFSD.A | 708.234 | 708.2453 | 0.0113 |
| | [7-12] | S.DADLAD.G | 641.23 | 641.2395 | 0.0095 |
| 2-16 | [7-11] | S.DADLA.D | 526.199 | 526.2125 | 0.0135 |
| | [8-12] | D.ADLAD.G | | | |
| 2-17 | [1-8] | SSGSFSDA.D | 779.275 | 779.2824 | 0.0074 |
| 2-18 | - | - | - | - | - |
| 2-19 | [9-17] | A.DLADGVSGG.E | 812.341 | 812.3402 | 0.0008 |
| | [9-18] | A.DLADGVSGGE | 941.380 | 941.3828 | 0.0028 |
| 2-20 | [7-17] | S.DADLADGVSGG.E | 998.397 | 998.4043 | 0.0073 |
| | [7-18] | S.DADLADGVSGGE | 1127.440 | 1127.4469 | 0.0069 |
| 2-21 | [1-12] | SSGSFSDADLAD.G | 1193.438 | 1193.4574 | 0.0194 |
| 2-22 | [1-11] | SSGSFSDADLA.D | 1078.420 | 1078.4305 | 0.0105 |
| | [1-17] | SSGSFSDADLADGVSGG.E | 1550.605 | 1550.6223 | 0.0173 |
| | [1-18] | SSGSFSDADLADGVSGGE | 1679.649 | 1679.6649 | 0.0159 |
| 2-23 | [1-18] | SSGSFSDADLADGVSGGE | 1679.646 | 1679.6649 | 0.0189 |

**B** Chromatograph of Peptide 3 (SSGSFSHADLADGVSGGE) and the peptides identified in the collected fractions

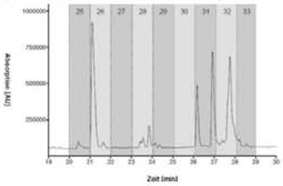


| Peptide – Fraction | AA | Sequence | Observed Mass *H$^+$ or (Na-H)$^+$ | Theoretical Mass *H$^+$ or (Na-H)$^+$ | Deviation (Da) |
|---|---|---|---|---|---|
| 3-25 | [1-9] | SSGSFHAD.L | 894.351* | 894.3593 | 0.0083 |
| 3-26 | [1-8] | SSGSFHA.D | 779.328* | 779.3324 | 0.0044 |
| | [2-8] | S.SGSFHA.D | 692.290 | 692.3004 | 0.0104 |
| 3-27 | - | - | - | - | - |
| 3-28 | [9-16] | A.DLADGVSG.G | 755.308 | 755.3188 | 0.0108 |
| | [9-18] | A.DLADGVSGE | 941.376 | 941.3828 | 0.0068 |
| | [6-17] | F.SHADLADGVSGG.E | 1085.484 | 1085.4863 | 0.0023 |
| 3-29 | [8-18] | H.ADLADGVSGG.E | 1012.410 | 1012.4199 | 0.0099 |
| | [6-18] | F.SHADLADGVSGGE | 1214.525 | 1214.5289 | 0.0039 |
| 3-30 | - | - | - | - | - |
| 3-31 | [1-11] | SSGSFHADLA.D | 1078.472* | 1078.4805 | 0.0085 |
| | [1-11] | SSGSFHADLAD.G | 1193.498* | 1193.5075 | 0.0095 |
| 3-32 | [1-17] | SSGSFSHADLADGVSGG.E | 1550.537* | 1550.6723 | 0.1353 |
| | [1-18] | SSGSFSHADLADGVSGGE | 1679.574* | 1679.7149 | 0.1409 |
| 3-33 | [1-18] | SSGSFSHADLADGVSGGE | 1679.714* | 1679.7149 | 0.0009 |

**C** Chromatograph of Peptide 4 (SSGSFSYADLADGVSGGE) and the peptides identified in the collected fractions

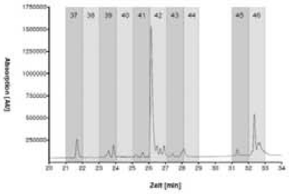


| Peptide – Fraction | AA | Sequence | Observed Mass (Na-H)$^+$ | Theoretical Mass (Na-H)$^+$ | Deviation (Da) |
|---|---|---|---|---|---|
| 4-37 | [1-5] | SSGSF.S | 506.176 | 506.1863 | 0.0103 |
| 4-38 | - | - | - | - | - |
| 4-39 | [9-16] | A.DLADGVSG.G | 755.310 | 755.3188 | 0.0088 |
| 4-39 | [9-17] | A.DLADGVSGG.E | 812.331 | 812.3402 | 0.0092 |
| 4-39 | [9-18] | A.DLADGVSGGE | 941.373 | 941.3828 | 0.0098 |
| 4-40 | - | - | - | - | - |
| 4-41 | [6-12] | F.SYADLAD.G | 776.299 | 776.3079 | 0.0089 |
| 4-41 | [1-8] | SSGSFSYA.D | 827.312 | 827.3188 | 0.0068 |
| 4-41 | [1-9] | SSGSFSYAD.L | 942.337 | 942.3457 | 0.0087 |
| 4-42 | [6-11] | F.SYADLA.D | 661.276 | 661.2809 | 0.0049 |
| 4-42 | [2-8] | S.SGSFSYA.D | 740.284 | 740.2867 | 0.0027 |
| 4-42 | [1-8] | SSGSFSYA.D | 827.317 | 827.3188 | 0.0018 |
| 4-43 | [6-17] | F.SYADLADGVSGG.E | 1133.458 | 1133.4727 | 0.0147 |
| 4-43 | [6-18] | F.SYADLADGVSGGE | 1262.501 | 1262.5153 | 0.0143 |
| 4-44 | [6-18] | F.SYADLADGVSGGE | 1262.503 | 1262.5153 | 0.0123 |
| 4-45 | [1-12] | SSGSFSYADLAD.G | 1241.484 | 1214.4938 | 0.0098 |
| 4-46 | [1-11] | SSGSFSYADLA.D | 1126.453 | 1126.4669 | 0.0139 |
| 4-46 | [1-18] | SSGSFSYADLADGVSGGE | 1727.680 | 1727.7013 | 0.0213 |

**Supplementary Figure 2: MS-assisted identification of meprin β cleavage sites *in vitro*.** (**A**–**C**) Chromatograms of peptide HPLC analysis from (Figure 2C) for CD99 WT (A), D92H (B), and D92Y (C). Collected fractions (numbered and shaded in gray) were subjected to MALDI MS/MS. Identified peptides are listed in the tables next to their corresponding peak numbers. For WT CD99 MALDI-MS confirmed the most abundant peak to belong to peptide SSGSFS.D corresponding to cleavage at 91S.92D in the full length protein (A). For CD99 D92H peptide SSGSFSHA.D was observed corresponding to cleavage at 93A.94D on CD99 (B), similarly for D92Y substitution the most predominant cleavage was between 8A.9D on the peptide or 93A.94D on WT CD99.

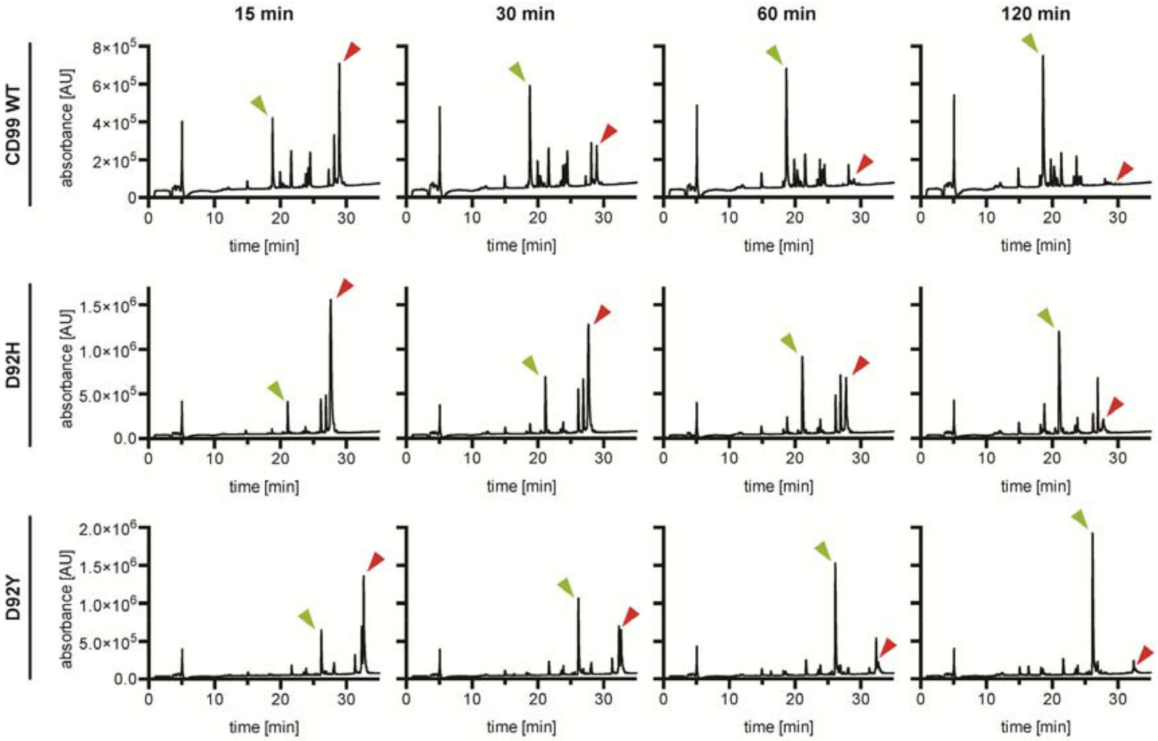


**Supplementary Figure 3:** ***in vitro*** **peptide cleavage kinetics.** Meprin β-mediated peptide cleavage was monitored time-dependently. Over time, peaks corresponding to the FL peaks from (Figure 2C) decreased (red arrowheads) while for each peptide one distinct peak corresponding to the main cleavage product increased (green arrowheads). The Y-axis scaling of the first graphs (time point 15 min) was applied to all graphs of the respective row.

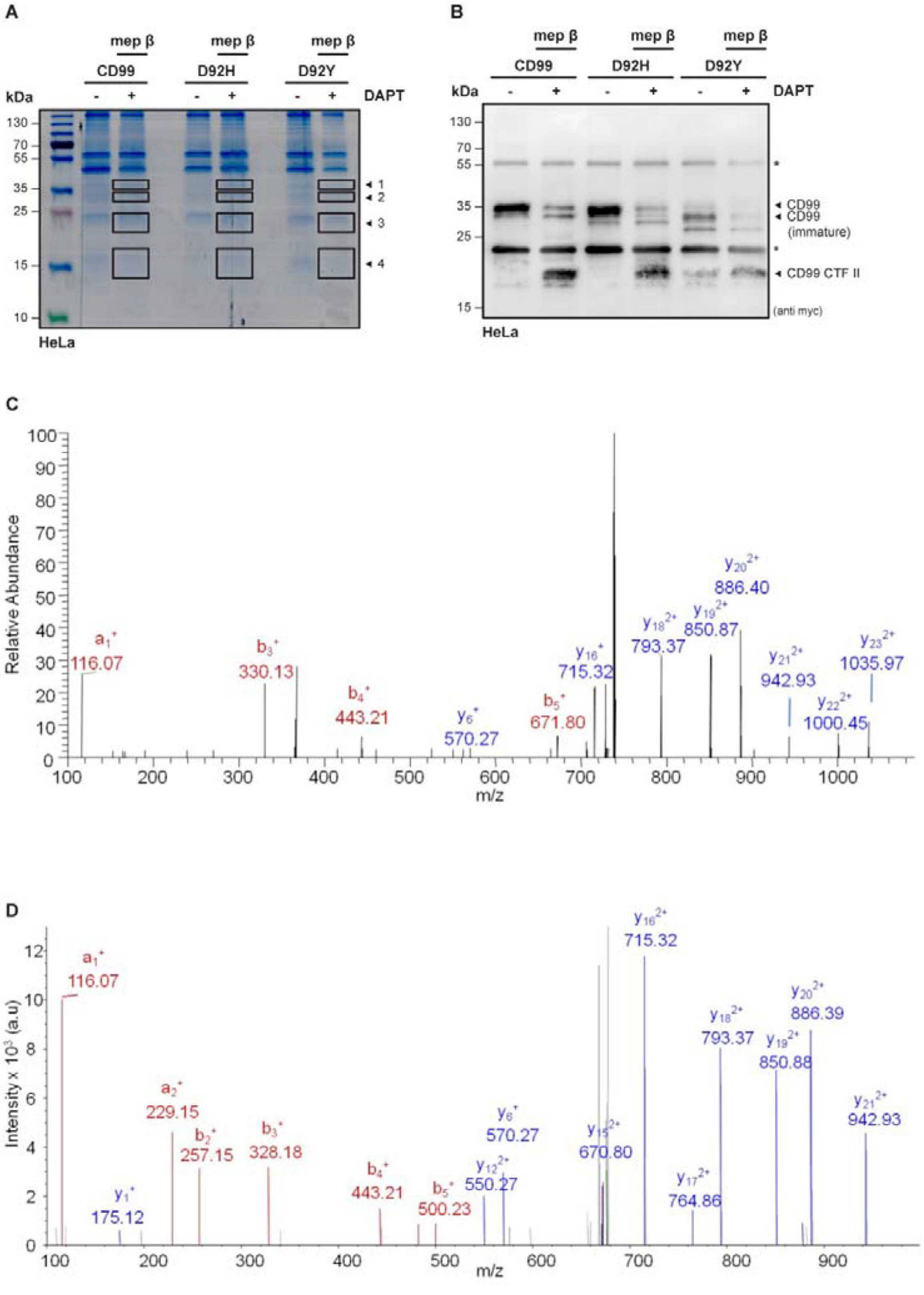


**Supplementary Figure 4: LC-MS assisted identification of meprin β cleavage sites in CD99 variants.** (**A**) Coomassie stained 12% SDS-PAGE gel loaded with immunoprecipitated CD99 C-term from cell lysates of HeLa cells that were previously transfected with CD99, D92H, or D92Y and meprin β and treated with γ-secretase inhibitor DAPT. Numbered boxes indicate gel parts that were cut out and subsequently analysed by LC-MS. (**B**) Successful immunoprecipitation of CD99 C-term was monitored by Western blot. (**C**) Band 4 for CD99 WT in the presence of meprin β and subsequent in-gel reductive dimethylation and digestion. MSMS spectra confirmed the identity of the cleavage site at 91S.92D with peptide sequence 91S.92D*ADLADGVSGGEGK*GGSDGGGSHR. Asterix denotes dimethylated amino acids. (**D**) Band 4 for CD99 D92H and CD99 D92Y in the presence of meprin β and subsequent in-gel reductive dimethylation and digestion. MSMS spectra confirmed the identity of the cleavage site at 93A.D94 with peptide sequence 93A.94D*LADGVSGGEGK*GGSDGGGSHR. Asterix denotes dimethylated amino acids.
